# Nucleated Red Blood Cells as Predictors of All-Cause Mortality in Cardiac Intensive Care Unit Patients: A Prospective Cohort Study

**DOI:** 10.1371/journal.pone.0144259

**Published:** 2015-12-29

**Authors:** José Gildo de Moura Monteiro Júnior, Dilênia de Oliveira Cipriano Torres, Maria Cleide Freire Clementino da Silva, Tadzia Maria de Brito Ramos, Marilene Leite Alves, Wellington Jorge Nunes Filho, Edgar Paulo Damasceno, Antônio Fernandes Brunet, Márcio Sommer Bittencourt, Rodrigo Pinto Pedrosa, Dário Celestino Sobral Filho

**Affiliations:** 1 Coronary Care Unit of PROCAPE (Pernambuco Cardiac Emergency Hospital), University of Pernambuco (UPE), Recife, Pernambuco, Brazil; 2 Laboratory of PROCAPE, University of Pernambuco, Recife, Pernambuco, Brazil; 3 Center for Clinical and Epidemiological Research, University of Sao Paulo, São Paulo, Brazil; University of Pittsburgh, UNITED STATES

## Abstract

**Background:**

The presence of nucleated red blood cells (NRBCs) in the peripheral blood of critically ill patients is associated with a poorer prognosis, though data on cardiovascular critical care patients is lacking. The aim of the present study was to assess the role of NRBCs as a predictor of intensive care unit (ICU) and in hospital all-cause mortality among cardiologic patients.

**Methods:**

NRBCs were measured daily in consecutive cardiac ICU patients, including individuals with both coronary and non-coronary acute cardiac care. We excluded patients younger than 18 years, with cancer or hematological disease, on glucocorticoid therapy, those that were readmitted after hospital discharge and patients who died in the first 24 hours after admission. We performed a multiple logistic analysis to identify independent predictors of mortality.

**Results:**

We included 152 patients (60.6 ± 16.8 years, 51.8% female, median ICU stay of 7 [4–11] days). The prevalence of NRBCs was 54.6% (83/152). The presence of NRBC was associated with a higher ICU mortality (49.4% vs 21.7%, P<0.001) as well as in-hospital mortality (61.4% vs 33.3%, p = 0.001). NRBC were equally associated with mortality among coronary disease (64.71% vs 32.5% [OR 3.80; 95%CI: 1.45–10.0; p = 0.007]) and non-coronary disease patients (61.45% vs 33.3% [OR 3.19; 95%CI: 1.63–6.21; p<0.001]). In a multivariable model, the inclusion of NRBC to the APACHE II score resulted in a significant improvement in the discrimination (p = 0.01).

**Conclusions:**

NRBC are predictors of all-cause in-hospital mortality in patients admitted to a cardiac ICU. This predictive value is independent and complementary to the well validated APACHE II score.

## Introduction

In healthy adults, peripheral blood is usually free of nucleated red blood cells (NRBCs) [1,2]. However, those cells may occur in some diseases, such as cancer, congestive heart failure, acute and chronic anemia and other hematological disorders [3,4]. Their presence in the peripheral blood has been associated with hypoxemia or infection in critical patients, owing to the high concentrations of erythropoietin, interleukin-3 and interleukin-6 [1,3,5–8] caused by local or systemic disorders, suggesting a reduction in oxidation of the tissue and/or inflammation.

Prior studies have also demonstrated that those cells may have significant prognostic implications, as their presence may occur in the three weeks prior to death [1–3]. In particular, Stachon et al. have demonstrated that NRBCs are a prognostic indicator in the Intensive Care Unit (ICU) environment, as its presence is associated with a higher in hospital mortality and higher ICU readmission rates, particularly when NRBC persist in the peripheral blood even after patients are clinically stable [1]. Although this has been demonstrated form general ICU patients, no data on patients admitted in the ICU for acute cardiovascular diseases exist. In the present study, we tested the hypothesis that the presence of NRBC may predict ICU (primary end-point) and in hospital (secondary end-point) all-cause mortality among patients admitted to a cardiac ICU.

## Materials and Methods

### Subjects and Protocol

All consecutive patients admitted in the cardiovascular ICU of the Pernambuco Cardiac Emergency Unit (PROCAPE), a specialized tertiary care cardiovascular teaching hospital with 250 beds, between May 2013 and January 2014 were included in the present study. This ICU is devoted to treat clinical patients with cardiovascular diseases. The study was approved by the Research Ethics Committee in the HOSPITAL COMPLEX HUOC/PROCAPE under number CAAE: 08412412.20000.5192 (Brazil Plataform). We excluded patients younger than 18 years, with cancer or hematological diseases, on glucocorticoid therapy, those that were readmitted after hospital discharge and patients who died in the first 24 hours after ICU admission. All patients included in the study signed a free and informed consent form.

The *Acute Physiology and Chronic Health Evaluation II* (APACHE II) and the *Sequential Organ Failure Assessment* (SOFA) scores were calculated from all patients twenty-four hours after admission to ICU, as previously described [9,10].

In the first twenty-four hours of admission, the patients were also classified as septic or not, according to previous criteria [11]. At the same time, the patients were also classified according to the cardiovascular disease etiology as coronary (acute or chronic) [12,13] or non-coronary (valvulopathies, perimyocardiopathies, cardiac arrhythmias), according to clinical and laboratorial and echocardiographic parameters.

### Laboratory tests

Blood samples were obtained daily in the morning until discharge from ICU. Blood parameters (NRBCs, leukocytes, neutrophils, hemoglobin and platelets) were measured using a Sysmex XE-2100 blood analyzer [14,15]. C-reactive protein was measured using a Roche Cobas Integra 400 analyzer.

For the NRBC measurement, we used the highest value during ICU admission for each individual. For the binary analysis, a positive NRBC was defined as any value above zero at any time during admission.

### Statistical analysis

All continuous variables are expressed as means ± standard deviation, or median and quartiles, as appropriate. Categorical variables are presented as absolute values and percents. Categorical variables were compared using two-tailed Pearson’s chi-squared (X^2^) test with the Yates correlation or Fisher’s exact test. The comparison of means, to establish the normality of the distribution, was carried out using the Kolmogov-Smirnov test, followed by Student’s t test for normal distribution variables or Mann-Whitney’s non-parametric test form non-normal distribution variables. The relative mortality risk was calculated for clinical and laboratory variables, with confidence intervals of 95%. Logistic univariate regressions were performed to evaluate predictors of mortality analyzing gender, age, APACHE score, SOFA score, sepsis diagnosis, presence of coronary artery disease and laboratorial data (Table 1). A multivariate logistic regression model (forward) was carried out to identify independent predictors of mortality. Variables with *P<0*.1 on univariate analysis were entered into a multivariate analysis. Due to the highly skewed distribution of the NRBC, we chose to perform its analysis as a binary variable based on the presence or absence of NRBC in the peripheral blood. In order to evaluate the incremental value of NRBC beyond clinical predictor of mortality in the ICU, we constructed ROC curves and calculated the area under the curve for each model, and compared a model including only the APACHE II score with a model including the APACHE II score and the NRBCs. The level of statistical significance adopted was p < 0.05. Sample size was calculated to assess a mortality odds ratio between patients with and without NRBC of 5.2 according to previous study by Stachon et al [1], assuming an α-error of 5% and a statistical power of 95%. The minimum sample size was 60 patients. Because many patients from our sample did not present sepsis criteria and NRBCs in peripheral blood, we decided to recruit more patients than previously calculated. Statistical analyses were conducted using the Statistical Program for Social Sciences (SPSS), version 10.0 for Windows.

**Table 1:** Biological and clinical characteristics of patients in intensive care according to NRBC status.

Characteristics	All patients	NRBC-positive (n = 83)	NRBC-negative (n = 69)	p
**Sex**				
Male	67 (44.1%)	40 (48.2%)	27 (22.0%)	0.263
Female	85 (51.8%)	43 (51.8%)	42 (78.0%)	
**Age (in years)**	60.6 ± 16.8	63.0 ± 16.1	57.6 ± 17.4	0.049^†^
**Skin color**				
White	59 (38.8%)	30 (36.1%)	29 (42.0%)	0.707
Mixed	67 (44.1%)	39 (47.0%)	28 (40.6%)	
Black	26 (17.1%)	14 (16.9%)	12 (17.4%)	
**Origin**				
Emergency	123 (80.9%)	65 (78.3%)	58 (84.1%)	0.568
Wards	22 (14.5%)	13 (15.7%)	9 (13.0%)	
CTRU	7 (4.6%)	5 (6.0%)	2 (2.9%)	
Median duration of stay in ICU (in days)*	7 (4; 11)	10 (5; 13)	4 (3; 7)	< 0.001^†^
**Mortality**				
ICU	56 (36.8%)	41 (49.4%)	15 (21.7%)	< 0.001^†^
Hospital	74 (48.7%)	51 (61.4%)	23 (33.3%)	0,001^†^
Median APACHE II*	21 (14; 27.5)	25 (19; 33)	15 (11; 22)	< 0.001^†^
**APACHE II**				
< 25 points	56 (36.8%)	39 (47.0%)	57 (82.6%)	< 0.001^†^
≥ 25 points	96 (63.2%)	44 (53.0%)	12 (17.4%)	
Median SOFA*	5 (2; 9)	8 (4; 11)	3 (1; 7)	< 0.001^†^
**SOFA score**				
< 7 points	86 (56.6%)	35 (42.2%)	51 (73.9%)	< 0.001^†^
≥ 7 points	66 (43.4%)	48 (57.8%)	18 (26.1%)	
**SEPSIS**				
Yes	84 (55.3%)	59 (71.1%)	25 (36.2%)	< 0.001^†^
No	68 (44.7%)	24 (28.9%)	44 (63.8%)	
**Coronary patient**				
Yes	74 (48.7%)	34 (41.0%)	40 (58.0%)	0.037^†^
No	78 (51.3%)	49 (59.0%)	29 (42.0%)	
**Coronary/Sepsis**				
Sepsis and coronary	30 (19.7%)	19 (22.9%)	11 (15.9%)	< 0.001^†^
Sepsis and non-coronary	54 (35.5%)	40 (48.2%)	14 (20.3%)	
No sepsis and coronary	44 (29.0%)	15 (18.1%)	29 (42.0%)	
No sepsis and non-coronary	24 (15.8%)	9 (10.8%)	15 (21.7%)	

NRBCs: nucleated red blood cells APACHE II: Acute Physiology and Chronic Health Evaluation II. SOFA: Sequential Organ Failure Assessment CTRU: Cardio-Thoracic Recovery Unit.

^†^ Statistically significant association.

*Median (P25; P75).

## Results

**Fig 1:**
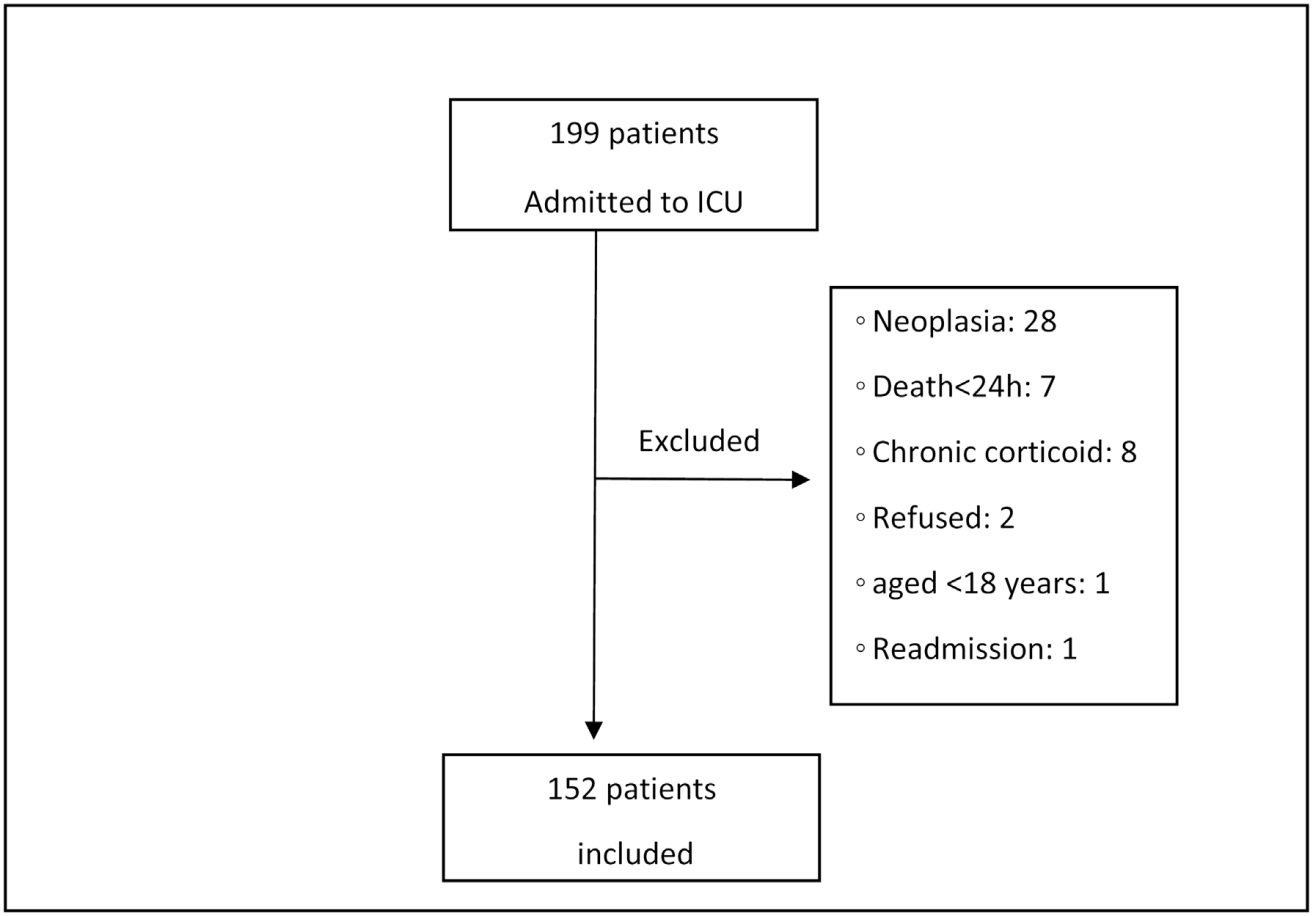
Flowchart of patients.

The study initially screened 199 patients, of whom 47 were excluded as shown in Fig 1. There were no follow-up losses and the final sample comprised 152 patients (60.6 ± 16.8 years, 51.8% female), with a median stay in ICU of 7 days (P25: 4; P75: 11) (Table 1). Approximately half of the sample presented NRBCs in the blood (83 patients; 54.6%) and the higher incidence of NRBC was noted after the ninth ICU day (Table 2). The presence of NRBCs was associated with older age, longer ICU stay, higher severity (SOFA and APACHE II) scores, sepsis and non-coronary cardiac etiology (Table 1). There was a negative correlation between levels of platelets and those of NRBCs (R = -0.25; p = 0.03). However, levels of C-reactive protein (R = 0.18; p = 0.14), leukocytes (R = 0.22; p = 0.06), neutrophils (R = 0.20; p = 0.09), and hemoglobin (R = -0.02; p = 0.87) were not associated with levels of NRBCs.

**Table 2:** Distribution of the measure of the maximum NRBC during the hospital stay in the ICU.

NRBC	Statistics
Sample	152 patients
NRBC maximum rating	
Zero	69 (45.4%)
From 1 a 100	50 (32.9%)
From 101 a 200	18 (11.8%)
> 200	15 (9.9%)
Presence of NRBC per day of hospitalization	
1° day	34/152* (22.4%)
2° day	36/124 (29.0%)
3° day	30/110 (27.3%)
4° day	20/77 (25.9%)
5° day	24/84 (28.6%)
6° day	16/57 (28.1%)
7° day	15/67 (22.4%)
8° day	10/46 (21.7%)
9° day	17/44 (38.6%)
After 9° day	11/35 (31.4%)

* Number of patients with positive NRBC / Number of patients with measures NRBC.

**Fig 2:**
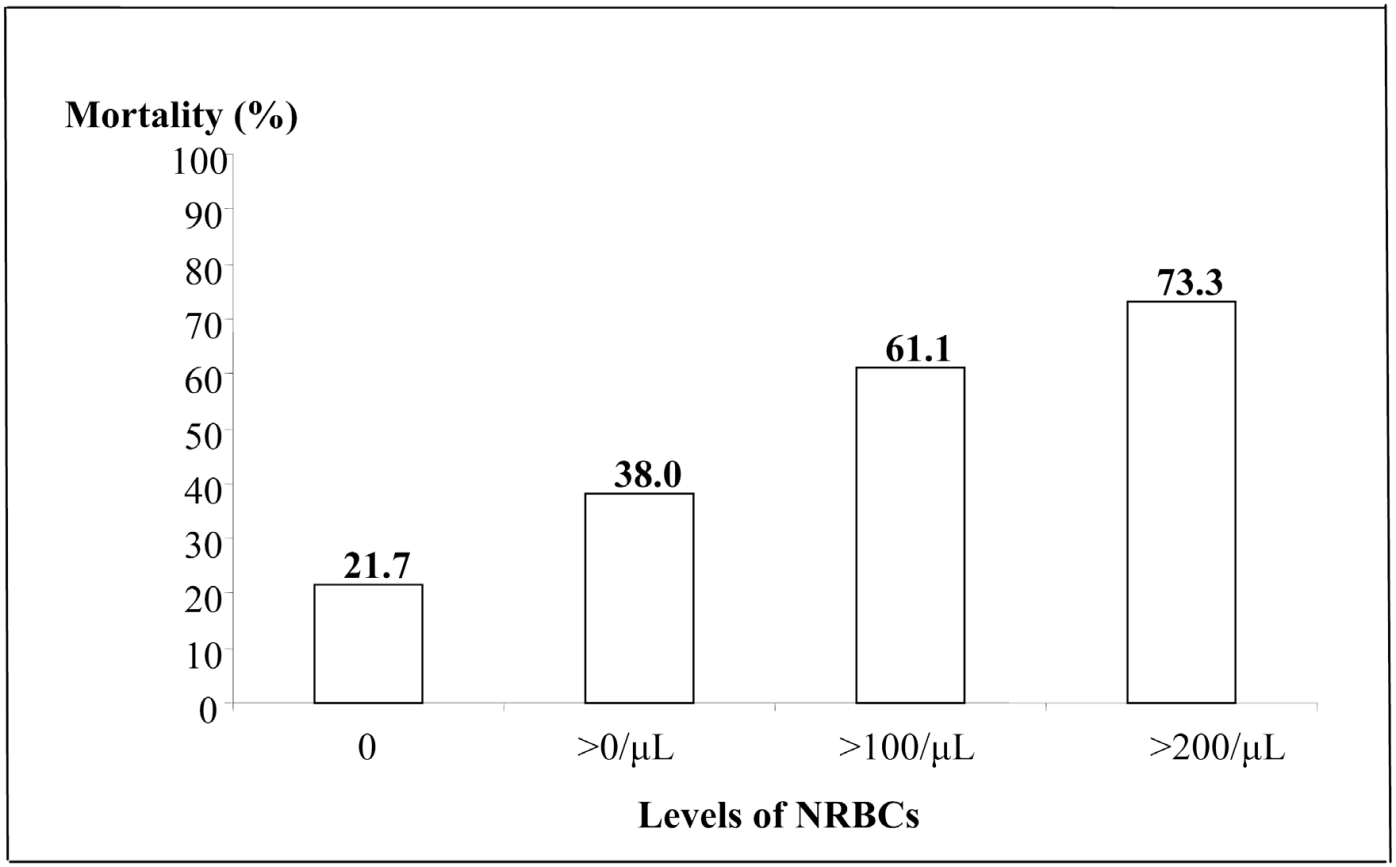
Mortality of ICU patients in relation to concentrations of nucleated red blood cells (NRBCs) P<0.001.

**Fig 3:**
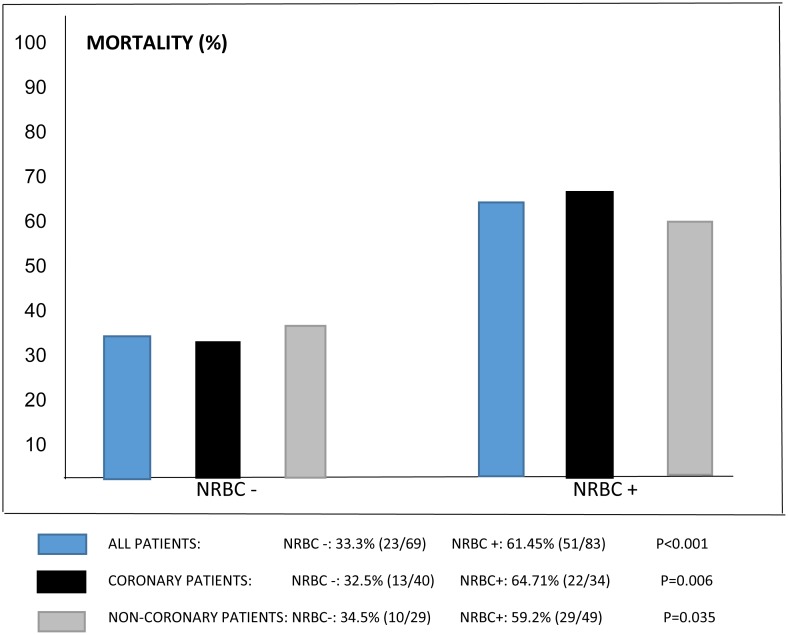
Mortality among coronary and non-coronary patients.

While the overall cardiovascular ICU mortality was 36.8% (56/152), increasing levels of NRBC were associated with a higher ICU mortality (Fig 2). Hospital mortality following discharge from ICU was 18.8% (18/96), with an in-hospital all-cause mortality of 48.7% (74/152). The presence of NRBC in the peripheral blood was associated with both ICU (49.4% vs. 21.7%, p<0.001) and in-hospital (61.4% vs. 33.3%, p = 0.001) all-cause mortality rates, respectively (Table 1) and was also associated with increased mortality among patients with coronary disease with 64.7% vs. 32.5% (OR 3.80; 95%CI: 1.45–10.0; p = 0.006), as well as among non-coronary disease patients with 59.2% vs 34.5% (OR 2.75; 95% CI: 1.06–7.15; p = 0.035) (Fig 3).

**Fig 4:**
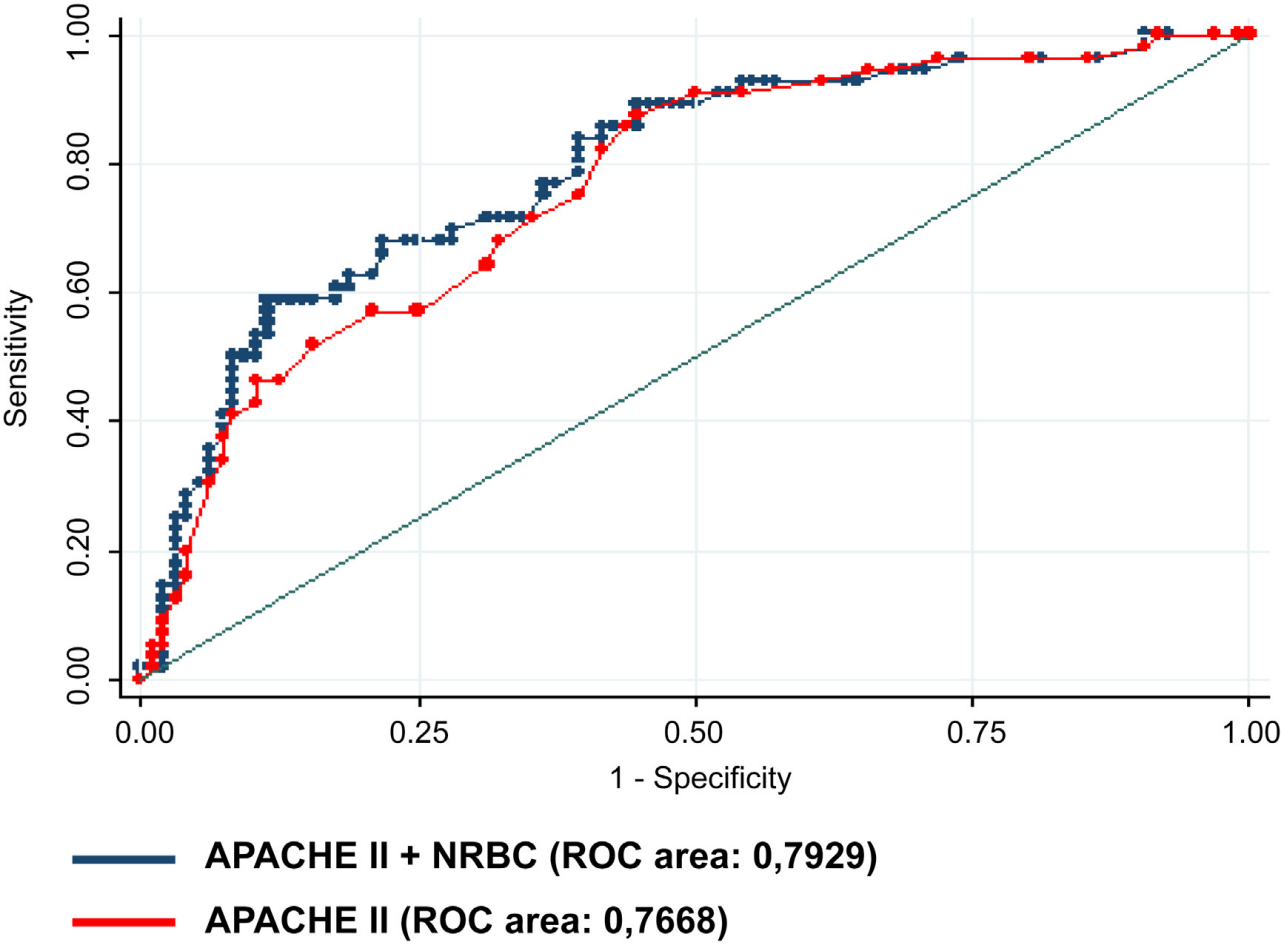
Comparison of ROC curves, p = 0.01.

After adjustment, for the clinical mortality risk using the APACHE II score, sex and sepsis, NRBC remained associated with all-cause in hospital mortality (OR 3.96; 95% CI: 1.45–10.8; p = 0.007) (Table 3). The inclusion of NRBC in a model already including the APACHE II score resulted in a significant increase in the area under the ROC curve from 0.7668 to 0.7929, p = 0.011 (Fig 4).

**Table 3:** Univariable and multivariable predictors of ICU mortality.

Variables	Univariate Model			Multivariate Model		
	OR	CI (95%)	P	OR	CI (95%)	P
**Biological**						
Sex						
Male	1.0	-	-	1.0	-	-
Female	2.07	1.06–4.03	0.034^†^	1.69	0.81–3.55	0.162
**Clinical**						
APACHE II Classification						
< 25 points	1.0	-	-	1.0	-	-
≥ 25 points	4.0	1.98–8.07	0.000^†^	2.41	1.08–5.37	0.031
SOFA Classification						
< 7 points	1.0	-	-	-	-	-
≥ 7 points	7.76	3.67–16.4	0.000^†^	-	-	-
Sepsis						
No	1.0	-	-			
Yes	3.37	1.64–6.89	0.001^†^	1.81	0.79–4.12	0.159
Coronary patient						
No	1.0	-	-	-	-	-
Yes	0.69	0.36–1.34	0.270	-	-	-
**Laboratory**						
NRBC^a^						
Zero	1.0	-	-	1.0	-	-
From 1 a 100	2.21	0.98–4.95	0.055	1.43	0.59–3.47	0.428
> 100	7,20	2.86–18.1	0.000	3.96	1.45–10.8	0.007
CRP^b^	1.015	0.997–1.034	0.100	-	-	-

OR: Odds Ratio. NRBC: nucleated red blood cells. APACHE II: Acute Physiology and Chronic Health Evaluation II. SOFA: Sequential Organ Failure assessment.

^a^ Divided into (0. 1–100. > 100).

^b^ Calculated risk of 5 unit increase.

^†^Statistical significance.

## Discussion

In the present study, we have demonstrated that the presence and levels of NRBC in the peripheral blood are independent predictors of all-cause in-hospital mortality for patients admitted to a cardiac ICU. This association also resulted in an important improvement in the discrimination beyond the well-validated APACHE II score.

Our findings corroborate previous results in patients admitted to general ICUs, suggesting that NRBCs are equally predictive of events in both the cardiac and non cardiac population. [1–7]. In this population, coronary pathologies were not found to be associated with mortality (OR 0.90; p = 0.739) and the presence NRBCs, as in general ICUs, was the best indicator of higher mortality (adjusted OR 3.24; CI (95%) 1.64–6.38; p = 0.001). Possible mechanisms involved in higher mortality among patients with elevated NRBC are hypoxemia and systemic inflammation [1,3,5–7]. Interestingly, our results have a much higher prevalence of NRBC (54.6%) then previous studies such as Stachon et al. [1] (17.5%), Desai et al. [3] (17.5%), Kuert et al. [5] (28.6%), and Shah et al. [7] (24.8%). The higher frequency of the presence of NRBCs in the present study may be explained by the overall higher severity of disease in our population, as expressed by the fact that 63.2% of the patients had an APACHE II score ≥25.

When the severity of disease, represented by the APACHE II score, is taken into account, the prevalence of our study closely replicate those previous studies, as in patients with APACHE II score ≥25, in whom the majority were NRBC-positive (53%). This concurs with the findings of Stachon et al. [1] and Desai et al. [3]. Moreover, the median SOFA score in this study was 8 in NRBC-positive patients and 3 in those who were NRBC-negative (Table 1). Desai et al. [3] found a median SOFA score of 10 among NRBC-positive and 8 among the NRBC-negative. There would therefore appear to be an association between the presence of NRBCs in the bloodstream and the seriousness of the condition of patients in ICU.

Patients who are candidates for discharge from a general ICU who present with NRBCs should be carefully evaluated, since they have a higher mortality rate [1]. The present study corroborates these findings and suggests a possible new use for measurement of NRBC levels in ICU, since the mortality of NRBC-positive patients discharged from ICU was 61.4%.

Our study must, however, be read within the context of its design. First, we have not split the coronary group into acute and chronic cases, which may have led to bias in analysis of this sub-group and influenced other laboratory variables, such as platelets [16–18]. Second, the small number of patients in extreme NRBC levels may limit our analysis. However, despite the relatively small sample size, the power for detecting differences in mortality among patients with and without NRBC was 92%. The estimates presented in the study are influenced by random error and from that standpoint may not be dependable.

In conclusion, NRBC predict all-cause mortality in patients admitted to a cardiac ICU. The predictive value of NRBC is independent and complementary to the prediction provided by the well validated APACHE II score.

## References

[1] Stachon A, Segbers E, Holland-Letz T, Kempf R, Hering S, Krieg M. Nucleated red blood cells in the blood of medical intensive care patients indicate increased mortality risk: a prospective cohort study. Critical Care 2007, 11(3): R62 10.1186/cc5932 . 17550592 PMC2206423

[2] Stachon A, Holland-Letz T, Krieg M. High in-hospital mortality of intensive care patients with nucleated red blood cells in blood. Clin Chem Lab Med 2004, 42(8):933–38. . 15387445 10.1515/CCLM.2004.151

[3] Desai S, Jones SL, Turner KL, Hall J, Moore LJ. Nucleated red blood cells are associated with a higher mortality rate in patients with surgical sepsis. Surgical Infections 2012, 13(6):360–5. 10.1089/sur.2011.089 . 23237100

[4] Danise P, Maconi M, Barella F, Palma AD, Avino D, Rovetti A, et al Evaluation of nucleated red blood cells in the peripheral blood of hematological diseases. Clin Chem Lab Med 2012, 50(2):357–60. 10.1515/CCLM.2011.766 . 22022981

[5] Kuert S, Holland-Letz T, Friese J, Stachon A. Association of nucleated red blood cells in blood and arterial oxygen partial tension. Clin Chem Lab Med 2011, 49(2):257–63. 10.1515/CCLM.2011.041 . 21118046

[6] Stachon A, Bolulu O, Holland-Letz T, Krieg M. Association between nucleated red blood cells in blood and levels of erythropoietin, interleukin-3, interleukin-6, and interleukin-12p70. Shock 2005, 24(1):34–9. . 15988318 10.1097/01.shk.0000164693.11649.91

[7] Shah R, Reddy S, Horst M, Stassnopoulos J, Lordan J, Rubinfeld I. Getting back to zero with nucleated red blood cells: following trends is not necessarily a bad thing. The American Journal of Surgery 2012 3; 203(3): 343–5. 10.1016/j.amjsurg.2011.10.002 . 22244074

[8] Roger HM, Xiaobing Y, Wen J, Smith R, Fibach E, Noguchi CT. Hypoxia Alters Progression of the Erythroid Program. Experimental Hematology 2008 1; 36(1):17–27. . 17936496 10.1016/j.exphem.2007.08.014 PMC2424406

[9] Knaus WA, Draper EA, Wagner DP, Zimmerman JE. APACHE II: a severity of disease classification system. Crit Care Med 1985 1; 13(10):818–29. . 3928249

[10] Vicent JL, Moreno R, Takala J, Willatts S, De Mendonça A, Bruining H, et al The SOFA (Sepsis—Related Organ Failure Assessment) score to describe organ dysfunction/failure. On behalf of the Working Group on Sepsis-Related Problems of the European Society of Intensive Care Medicine. Intensive Care Med 1996 7; 22(7):707–10. . 8844239 10.1007/BF01709751

[11] Dellinger RP, Levy MM, Rhodes A, Annane D, Gerlach H, Opal SM, et al Surviving Sepsis Campaign: Internacional Guidelines for Management of Severe Sepsis and Septic Shock 2012. Intensive Care Med 2013 2; 39(2):165–228. 10.1007/s00134-012-2769-8 . 23361625 PMC7095153

[12] Hamm CW, Bassand JP, Agewall S, Bax J, Boersma E, Bueno H, et al ESC Guidelines for the management of acute coronary syndromes in patients presenting without persistent ST-segment elevation: The Task Force for the management of acute coronary syndromes (ACS) in patients presenting without persistente ST-segment elevation of the European Society of Cardiology (ESC). European Heart jornal 2011 12; 32(23):2999–3054. 10.1093/eurheartj/ehr236 . 21873419

[13] Fraker TD, Fihn SD, Gibbons RJ, Abrams J, Chatterjee K, Daley J, et al 2007 Chronic angina focused update of the ACC/AHA 2002 Guidelines for the Management of patients with Chronic Stable Angina: a Report of the American College of Cardiology/American Heart Association Task Force on Practice Guidelines Writing Group to Develop the Focused Update of the 2002 Guidelines for the Management of Patients with Chronic Stable Angina. Circulation 2007 12 4; 116(23): 2762–72. . 17998462 10.1161/CIRCULATIONAHA.107.187930

[14] Nakul-Aquaronne D, Sudaka-Sammarcelli I, Ferrero-Vacher C, Starck B, Bayle J. Evaluation of the Sysmex Xe-2100 hematology analyzer in hospital use. Journal of Clinical Laboratory Analysis 2003; 17(4): 113–23. . 12784259 10.1002/jcla.10083 PMC6807756

[15] Pipitone S, Pavesi F, Testa B, Bardi M, Peri GB, Gennari D, et al Evaluation of automated nucleated red blood cells counting on Sysmex XE5000 and Siemens ADVIA 2120. Clin Chem Lab Med 2012 10 1; 50(10):1857–9. 10.1515/cclm-2012-0148 . 23089720

[16] Núñez J, Núñez E, Bodí V, Sanchis J, Mainar L, Miñana G, et al Low lymphocyte count in acute phase of ST-segment elevation myocardial infarction predicts long-term recurrent myocardial infarction. Coronary Artery Disease 2010 1; 21(1):1–7. . 20050312 10.1097/mca.0b013e328332ee15

[17] Azab B, Zaher M, Weiserbs KF, Torbey E, Lacossiere K, Gaddam S, et al Usefulness of neutrophil to lymphocyte ratio in predicting short- and long-term mortality after non-ST elevation myocardial infarction. The American Journal of Cardiology 2010 8 15; 106(4): 470–6. 10.1016/j.amjcard.2010.03.062 . 20691303

[18] Labriolle AD, Bonello L, Lemesle G, Roy P, Steinberg DH, Xue Z, et al Decline in platelet count in patients treated by percutaneous coronary intervention: definition, incidence, prognostic importance and predictive factors. European Heart Journal 2010 5; 31(9): 1079–87. 10.1093/eurheartj/ehp594 . 20089516

